# Suits for Malpractice in Maine

**Published:** 1878-08

**Authors:** 


### Suits for Malpractice in Maine.

At the recent meeting of the Maine Medical Association at Portland, Dr. E. F. Sanger, of Bangor, presented the following statistics: "There are about six hundred regular physicians in the state. I have received reports from one hundred and fourteen of them, and in these reports have selected seventy cases of malpractice-suits, fifty-five threats, and fifty-eight who have never been sued or threatened. Many, from modesty and a disinclination to advertise their contributions to the patients and attorneys who follow us as the shark does the emigrant ship, have failed to report. The new-beginners and unthrifty of our profession are exempt from these rapacious attacks, and yet I have reports of more than one hundred and twenty-five cases of such suits and threats within the remembrance of the present generation. Excluding those who will not report and those who would naturally be exempt from such attacks, we safely may say that hardly a surgeon of experience and property, in active practice, escapes the annoyance and expense of these vexatious attacks. Of the seventy who were sued for damages of from $1,000 to $25,000, six paid from $100 to $350 rather than stand trial, and nine were cast in damages for malpractice of from $103 to $2,000 The nine plaintiffs who compounded were not worth any thing. Eight of the nine who got damages were not worth a dime, and all but six of those who failed to sustain their suits were worthless. Out of seventy prosecutions only seven were able to pay taxable costs, and very many of them were drunken and shiftless persons. One in eight got a verdict for damages. The nine who settled got $2,980; but as one had six trials and two others one each, and as none of the plaintiffs were able to pay the costs of a suit, it is probable that lawyers and witnesses absorbed the entire amount The total amount paid upon the verdict for damages was about $6,500, as many of the suits were long and expensive, and it is probable that more than one-half of the amount was absorbed in expenses, leaving about $3,000 to be distributed. How much the lawyers realized, and how much the patients, does not appear; but a very small amount went to fill hungry mouths."—*Louisville Medical News.*

